# 

**Published:** 1870-07

**Authors:** 


### BOOKS, MAGAZINES AND PERIODICALS.

PETERSON'S MAGAZINE.—This excellent Ladies' Magazine for June has a beautiful steel engraving, entitled "Something of a Flirt," the usual number of fashion plates, many patterns and devices of interest to ladies, and many excellent contributions of stories, &c. Charles J. Peterson, 306 Chestnut St., Phila., Publisher.—Price only $2.00 a year.

THE LITTLE CORPORAL MAGAZINE, for Boys and Girls, is the very best juvenile magazine published. It employs the best and most entertaining writers for children, in the country, and furnishes more valuable reading matter for one dollar a year, than any other magazine. It is published monthly, by Sewell & Miller, Chicago, Ills.

MRS. GLEASON'S BOOK.—Many books have been attempted upon the diseases peculiar to women, but until the appearance of "Talks to My Patients," by Mrs. R. B. Gleason, M. D., nothing has been written that completely exhausts the subject, and leaves a book in the hands of our wives and daughters worthy to be read by them. It is just such a book that every mother should place in the hands of her grown daughter. It will teach her just what is necessary to be known, and no more. It is written in plain, chaste and intelligible words, and is an honor to the head and heart of the lady who wrote it. We earnestly advise every mother and daughter to read it.

It is for sale by Hall Brothers of this city, price $1.50, and sent by mail, postage paid, on receipt of price.

ZELL'S ENCYCLOPEDIA.—This valuable publication has completed its first volume. Thirty-one numbers have now been issued and have given universal satisfaction to the subscribers of the work. The publishers guarantee that the work shall not cost more than $25.00 when complete. It will be by far the handsomest, cheapest, and most thorough and complete gaziteer and dictionary in print. T. Ellwood Zell, publisher, 17 and 19 South 6th Street, Philadelphia.

"HEALTH BY GOOD LIVING," is the title of a book written by W. W. Hall, M. D. The Doctor contends that good health can be maintained and common diseases *cured* by eating the best of meats, fish, poultry, game, fruits, &c.—The book is well worth reading, and contains many useful hints upon matters of health. Price $1.50. For sale by Hall Bros.

"PHYSICAL LIFE OF WOMAN." The fifth edition of this excellent work, by Dr. Napheys, has been issued, an evidence of its popularity and usefulness. It is not only addressed to the maiden and maton, but should also be read by the husbands. It treats in a plain and familiar manner of the diseases common to women. Price $2.00, sent to any address, post paid, on receipt of price, by Hall Brothers.

TWO NEW BOOKS.—The two popular books now being read by the American people, are entitled "Hedged In," by the author of "Gates Ajar," and "The Old Fashioned Girl," by the author of "Little Women." Both these books are meeting with an immense sale, and are being universally read. Price of each, $1.50. For sale by Hall Bros.

THE HERALD OF HEALTH, a journal of physical culture, has been received. We notice among the contributors, the names of Henry Ward Beecher, Mrs. Elizabeth Oakes Smith, Prof. Huxley, Dr. Wood, and other noted writers. It is a journal calculated to do much good and is deserving of the large circulation which it enjoys. Wood & Holbrook, publishers, at 13 and 15 Laight St., New York. Price $2.00 per annum.

W. D.—*Parkville, Mo.*—The Doctor mentioned is not reliable. Never trust a "Doctor" who sends you circulars and solicits your patronage.

SUBSCRIBER—*Waverly, N. Y.*—The disease mentioned is curable.

Dr. J. L. J.—*Dyer Station, Tenn.*—You can obtain an Ophthalmoscope of Geo. Tiemann & Co., N. Y. You will find their address in the BISTOURY. A case of eye instruments can be had at same place.

"Stellwag on the Eye" is the best work on the subject. Wm. Wood & Co., Pub., N. Y.

Dr. G.—*Sandwich, Ill.*—Use the following prescription for an adult. You will have the satisfaction of bringing the worm away entire; R. Olei terebinth.; Olei ricini, *aa* f, dr. ij; Spts. lavendulæ comp., f. dr. ij. M. Give it at one dose, in the morning, after 24 hours fasting. We have employed it very often in tape worm, and have never known it to fail.

Mrs. W. N.—*Williamsburgh, Va.*—We cannot go so far for so small a fee. Apply to some surgeon nearer you.

P. L. R.—*Jamestown, N. Y.*—Apply to Dr. Wm. H. Gregg, of this city. He is a practical chemist and will make the analysis for you.

T. S.—*Harrisburg, Pa.*—Procure a nasal douche and use it morning and evening, with a solution of salt—teaspoonful to the pint of warm water. This is the best local application you can make yourself for catarrh.

L.—*Troy, N. Y.*—Have it removed surgically by all means. Do not apply a cancer plaster about your face or eye—it is a very dangerous and hazardous proceeding.

